# Evaluation of scientific output in Dentistry in Spanish Universities

**DOI:** 10.4317/medoral.21656

**Published:** 2017-06-18

**Authors:** María De la Flor-Martínez, Pablo Galindo-Moreno, Elena Sánchez-Fernández, Ernest Abadal, Manuel-Jesús Cobo, Enrique Herrera-Viedma

**Affiliations:** 1 Department of Oral Surgery and Implant Dentistry, University of Granada (Spain); 2 Department of Library and Information Science, University of Barcelona (Spain); 3 Department of Computer Science, University of Cadiz (Spain); 4 Department of Computer Science and Artificial Intelligence, University of Granada (Spain)

## Abstract

**Background:**

The aim of this study was to assess the scientific output of Spanish universities that offer a bachelor’s degree in dentistry through the use of various bibliometric indicators.

**Material and Methods:**

A total of 21 universities offered a bachelor’s degree in dentistry in academic year 2016-2017. The search for papers published by authors associated with these institutions was carried out using the selection of journals listed in the Journal Citation Reports (JCR) and the Web of Knowledge database for the period 1986-2017. On the basis of these data, we determined the output, the h-, g- and hg-indexes, the most productive authors, international collaborations, and the most relevant journals.

**Results:**

Public universities obtained better results than private universities. The University of Valencia was ranked first, followed by the Complutense University of Madrid and the University of Granada. The most productive author was José Vicente Bagán, but the author with the highest h-index was Mariano Sanz and Manuel Toledado. The universities with the greatest output and highest citation rates had more international collaborations. The most developed fields in Spanish universities were Oral surgery, Oral medicine and Dental materials. The universities had different models of production. At universities such as Barcelona or Valencia, the production was focused on very few departments and authors. At the other extreme, the University of Granada had various sources of research and authors, which meant that its output and citation rate could increase more.

**Conclusions:**

University faculties must provide suitable academic and research training, and therefore must be assessed using objective criteria and bibliometric tools. Although the number of university schools and faculties that teach dentistry has increased, and particularly the number of private universities, there is no correlation between their quality and output and the number of places offered on their courses.

** Key words:**Dentistry, h-index, impact factor, universities, Spain.

## Introduction

In recent decades, the area of Dentistry has developed considerably in terms of scientific output and citation rate, which now equal those in other areas of Medicine. A number of bibliometric studies have evaluated dentistry in an international context (1), whilst others have focused on specific countries. Brazil is one of the countries that have been most widely studied (2-4). Various bibliometric indicators have been applied to specific dentistry journals (5), or to subjects such as periodontics (6,7) or orthodontics (8).

In Spain, there has been a change in scientific output and citation rate, as shown in various bibliometric studies, some of which are general (9), whilst others are focused on the fields of biomedicine (10-13), primary care (14), autism (15), cardiology (16), or dentistry (17) .

Dentistry in Spain is a young discipline that was separated from Medicine and established as an independent degree in 1986. Since then, the number of Schools offering a degree in Dentistry has risen, which has led to higher numbers of dentistry graduates, particularly those with qualifications from private universities.

At the same time, various indicators for assessing science have been designed in the field of bibliometrics. These include the well-known journal Impact Factor (18) and the h-index (19), which has become one of the most popular indicators since it emerged in 2005. The h-index has some limitations, but is complemented by other indexes, such as the g-index (20), which is based on assessing papers with high citations rates, and the hg-index (21) .

The aim of this study was to assess the scientific output of Spanish universities that offer a degree in dentistry, and analyse the most productive authors. We carried out a quantitative (based on the number of papers published) and qualitative evaluation (through the application of the h-, g- and hg-indexes) of universities. We identified the most productive authors, and those with the highest h-index. We also analysed these authors’ collaborations and the journals in which they published.

## Material and Methods

A total of 21 (12 public and 9 private) Spanish universities offered a bachelor’s degree in dentistry in academic year 2016-2017, according to the Ministry of Education, Culture and Sport’s website.

We devised a search strategy to find papers published by authors from these universities. We looked in journals listed in JCR 2015, and took into account changes in the name of the journals and journal supplements. Changes in the name of journals and in the ISSN, along with special journal supplements (7), were identified using the information provided in the Ulrich and Pubmed databases. Each university was analysed individually according to its name and abbreviation.

The study period was from 1986, the year in which Dentistry was first approved as an independent degree to Medicine in Spain, until 2017. The Web of Knowledge and other databases were consulted in 2017 January 3rd. To identify all of the most productive authors, different forms of names were used as well as the ORCID identifier, when applicable.

The data were processed by the database itself, and the h-, g- and hg-indexes were obtained by downloading the information and processing it using Microsoft Excel version Windows 2010.

## Results

**Figure 1:**
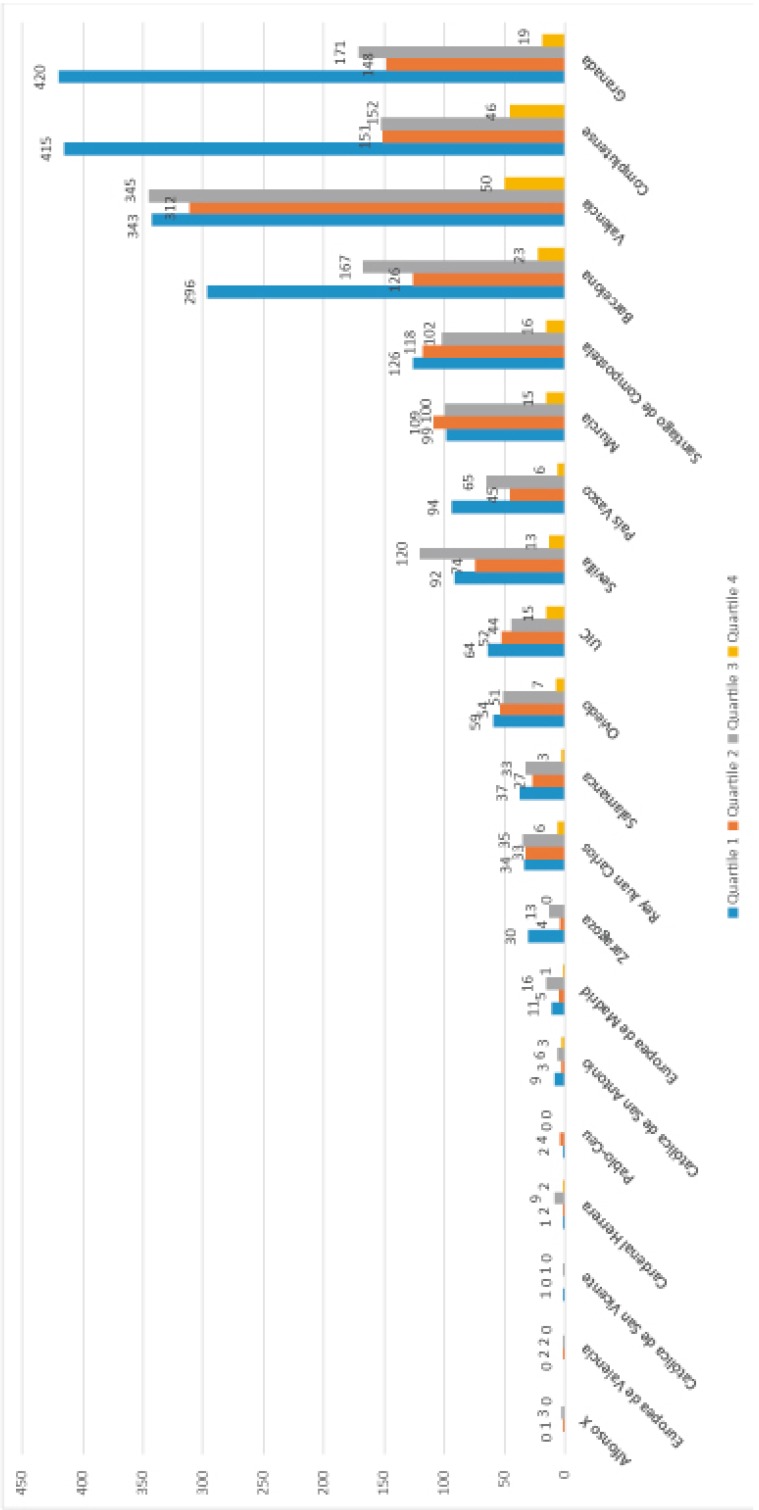
Representation of universities by number of publications and quartile.

The universities of Valencia, Complutense and Granada were ranked highest in quantitative (number of documents) and qualitative (h-index) terms. Private universities were ranked lowest on scientific output and bibliometric indexes, except the International University of Catalunya, which was in tenth place in the table, above the Rey Juan Carlos and Zaragoza public universities ( Table 1). Although the University of Granada was ranked third, it had the highest number of publications in the first quartile of the JCR (Fig. 1). The relative importance of dentistry in the various universities was low, and generally did not reach 1% of the total output. Notably, Dentistry accounted for 17.72% of the total output at the International University of Catalunya ( Table 1).

**Table 1:**
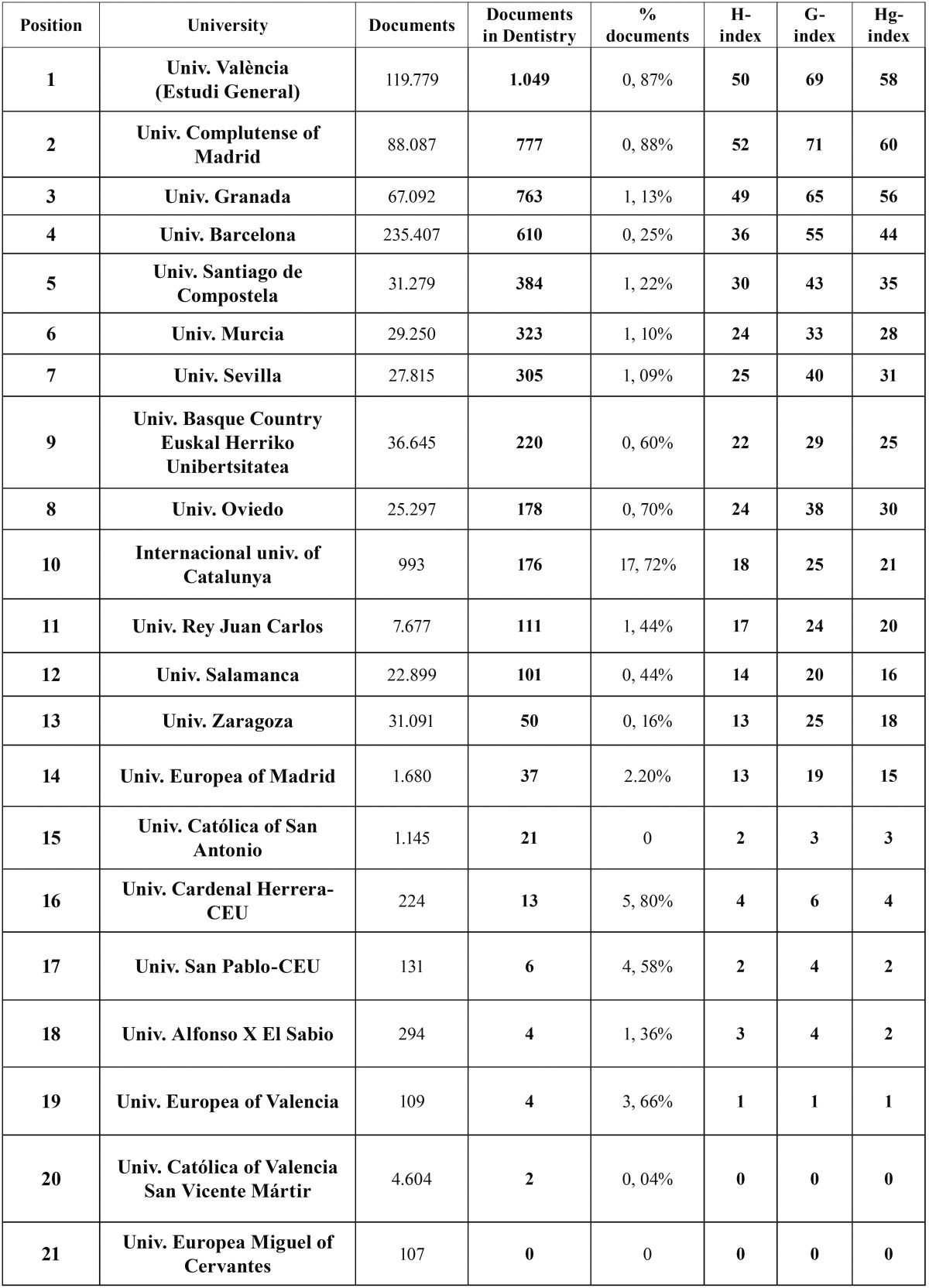
Scientific output of Spanish universities.

We only selected authors who had published at least 60 documents in 1986-2017, which resulted in a total of 32 authors ( Table 2). The most productive author was José Vicente Bagán (University of Valencia) with a total of 280/278 WoS and Scopus documents respectively, followed by Cosme Gay Escoda (University of Barcelona) with 267/264 WoS and Scopus documents respectivaly, and Manuel Toledano (University of Granada) with 270/246 WoS and Scopus documents. However, a qualitative analysis of the h-index placed Manuel Toledano in first position with an h-index of 41 in Wos and Scopus, and Mariano Sanz (h-index of 38 WoS and 43 Scopus), Raquel Osorio (University of Granada) in third position with 38/36 WoS and Scopus. Only eight women were on the list (25% of the total).

**Table 2:**
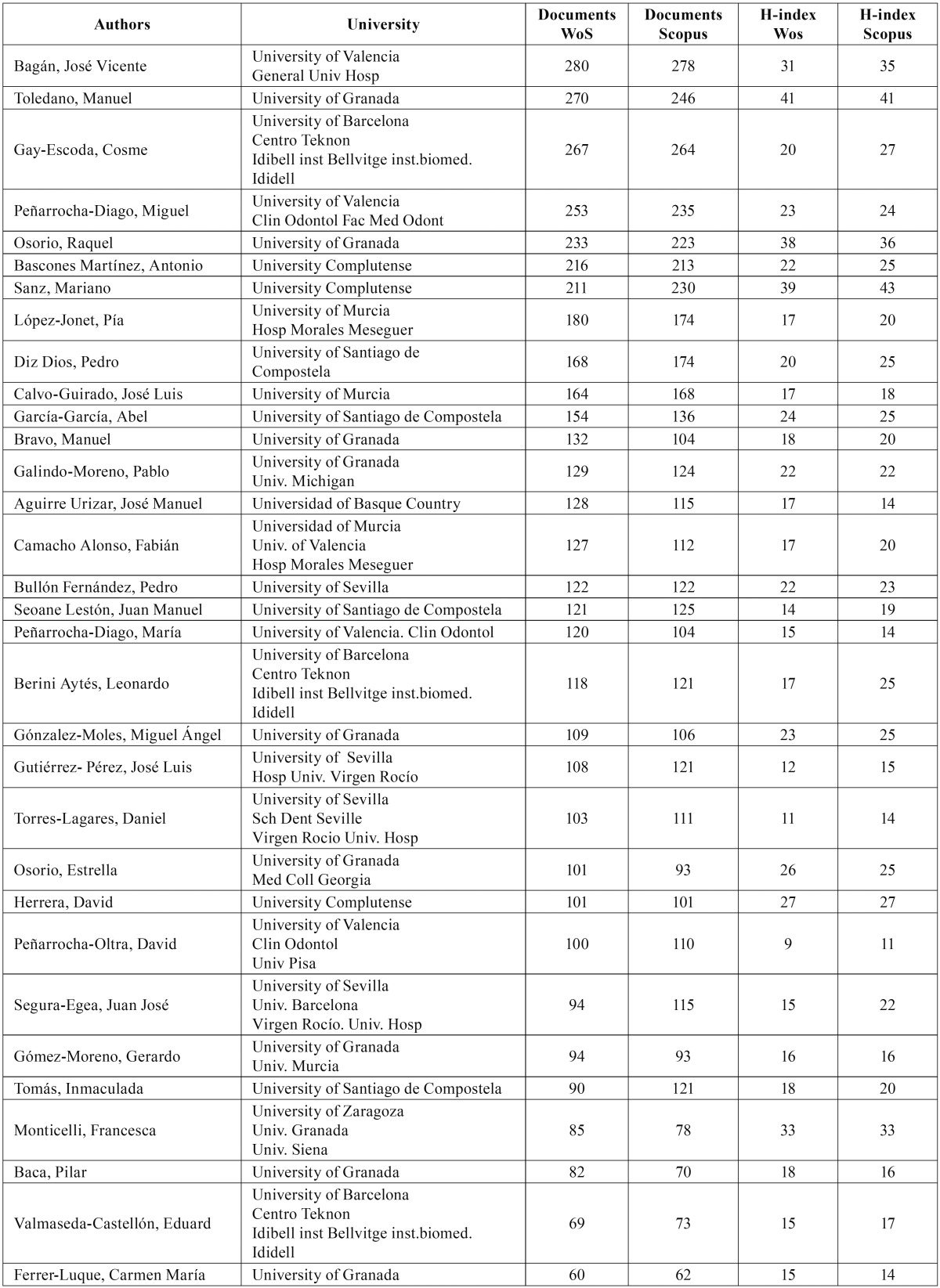
Scientific output of Spanish authors on dentistry.

The University of Granada had the highest number of researchers (nine;  Table 3) and carried out research in various fields (oral surgery, dental materials, endodontics, oral medicine and preventive dentistry). Other universities had a maximum of four authors on the list and their research was focused on specific departments.

**Table 3:**
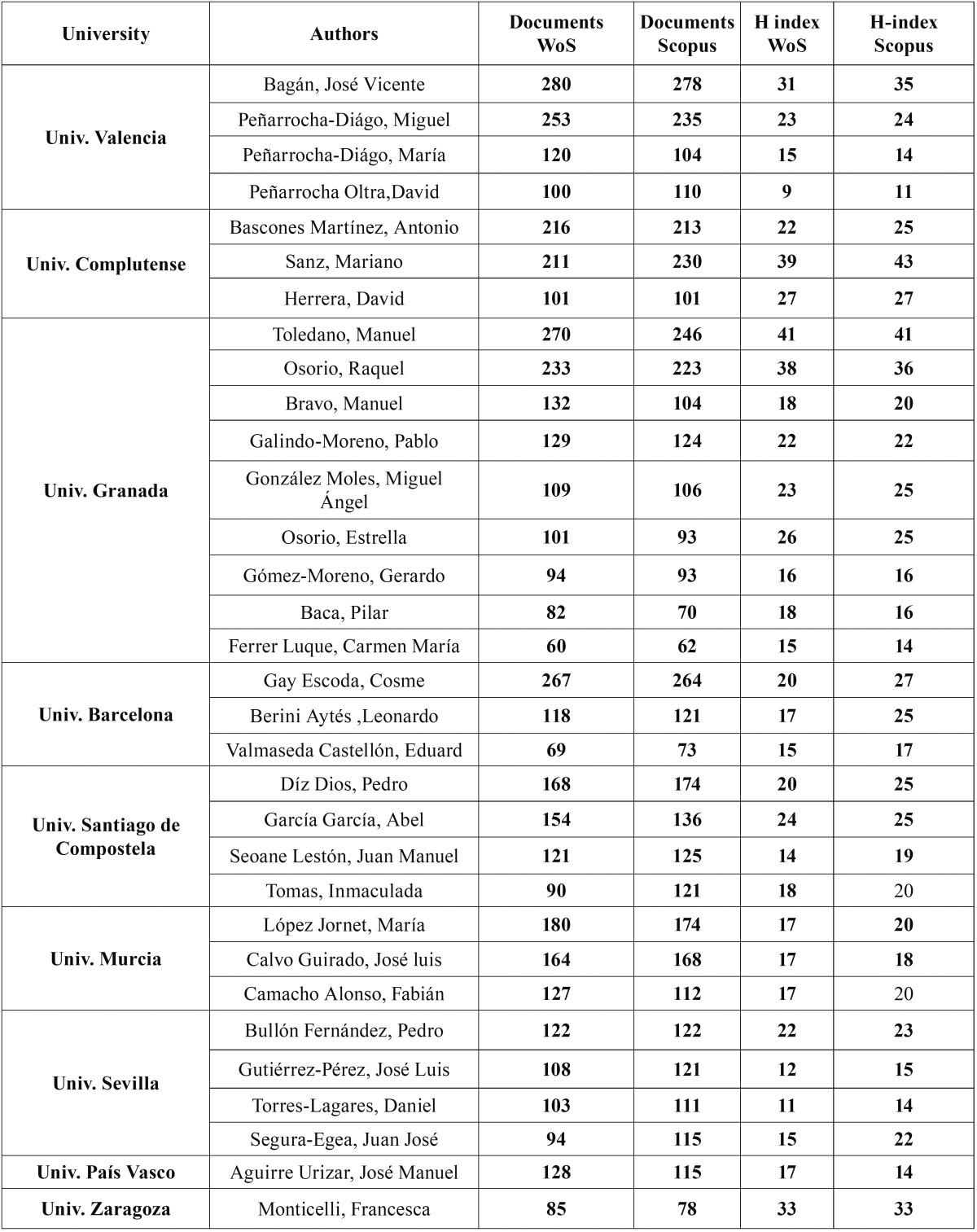
Distribution of selected authors by universities.

The universities of Valencia, Granada and Barcelona, as well as the Complutense University of Madrid, had the highest output and index values, and the highest number of international collaborations ( Table 4- Table 7).

**Table 4:**
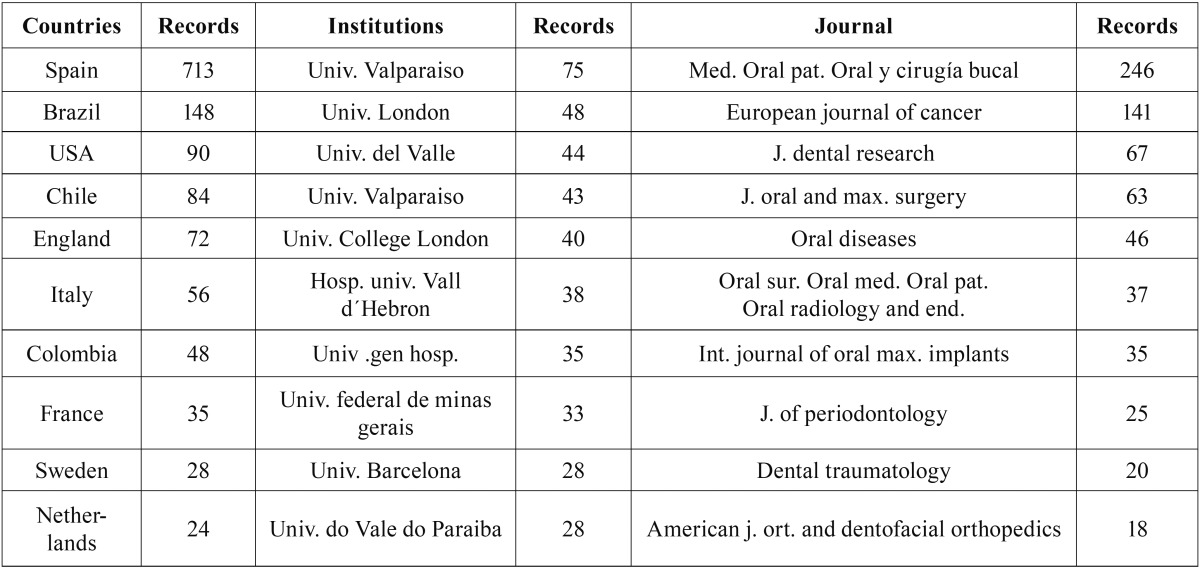
Assessment of the University of Valencia, according to international collaborations by country, institution and journal.

**Table 5:**
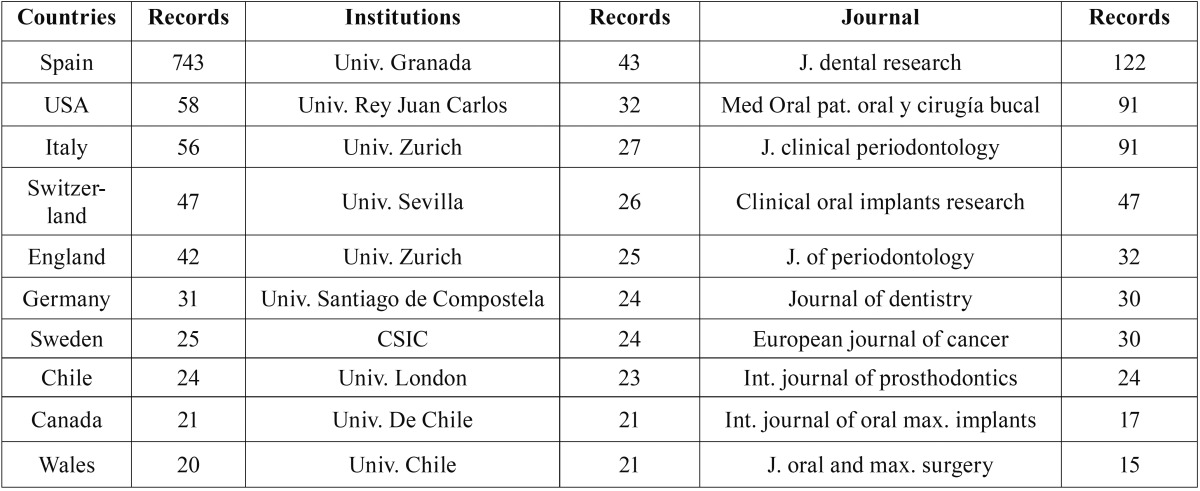
Assessment of the Complutense University of Madrid, according to international collaborations by countries, institution and journal.

**Table 6:**
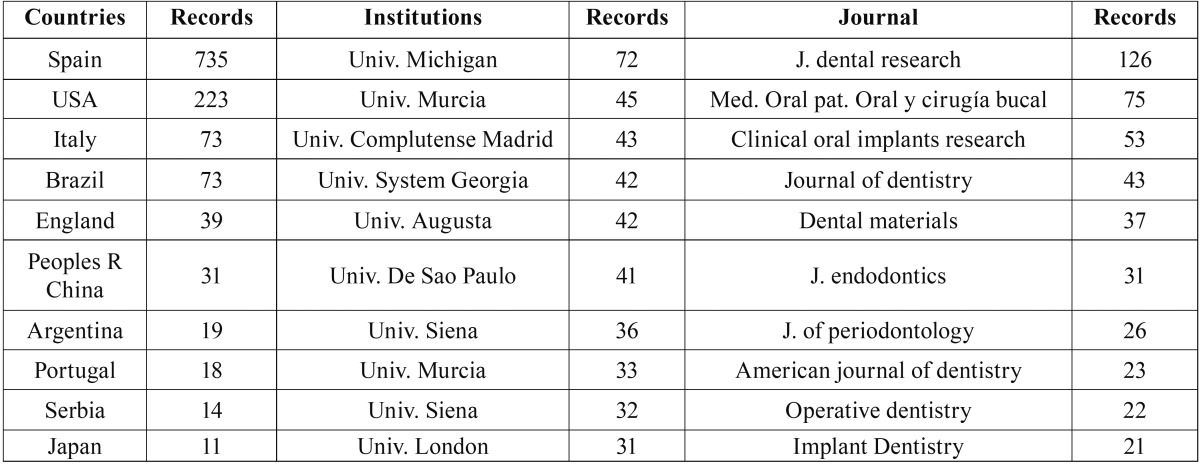
Assessment of the University of Granada, according to international collaborations by country, institution and journal.

**Table 7:**
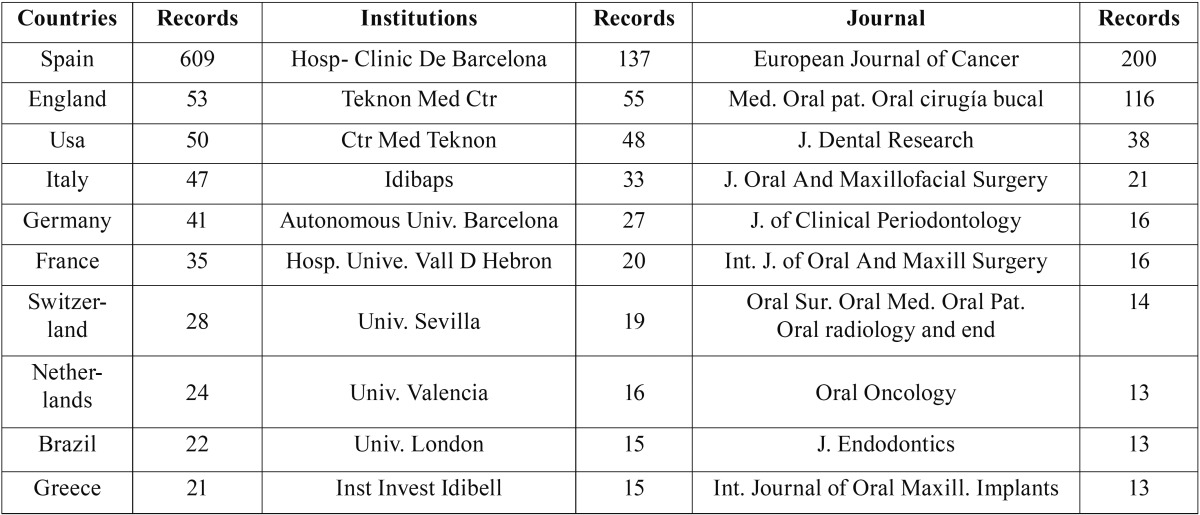
Assessment of the University of Barcelona, according to international collaborations by country, institution and journal.

## Discussion

Scientific output in Spain has increased in all disciplines (9). A study on biomedicine in Spain classifies organisations into five sectors (13): the university sector (comprised of universities, university institutions and scientific technical services), the health sector (comprised of hospitals and primary care centres), public research organisations (PRO) (comprised mainly of the Spanish National Research Council [CSIC] and the Carlos III Health Institute [ISCIII]), the business sector (comprised of pharmaceutical companies), and government departments and organisations (in which associations also have an influence). Most dentistry activity is private. Very little dentistry activity takes place in centres or hospitals, where it is generally associated with the oral and maxillofacial surgery department. It is difficult to identify centres and institutions that are associated with universities in this field, as affiliations are not standardised. Therefore, in this study we only evaluated universities recognised by the Ministry of Education, Culture and Sports for academic year 2016-2017. This was considered to be the most objective criterion for assessing the Spanish education system. However, the disadvantage is that some of the main hospitals and health centres were not included, such as the Hospital de la Fe associated with the University of Valencia, or the Teknon or Bellvitge hospitals in the case of the University of Barcelona.

The institutions with the highest scientific output and h-, g- and hg-indexes were public universities. These results are similar to those of studies carried out on other disciplines in Spain. They are related to the education model in Spain, in which the public sector plays a more important role than the private sector (9), unlike models in other countries such as the USA.

In general, there was little variation in the quantitative (number of documents) and qualitative (h-index) rankings, unlike data gathered on the international situation (7). The universities at the top of the ranking were those with the greatest degree of international cooperation.

Output in dentistry has increased in recent years. However, unlike other fields, the greatest development has been in journals focused on different areas, rather than in journals specific to this field. This could explain why authors such as Manuel Toledano, Raquel Osorio and Francesca Monticelli, who are specialised in dental materials (22), have the best indexes. These fields are more frequently cited than areas such as dentistry. This result is related to the premises that are required and imposed by the National Agency for Quality Assessment and Accreditation of Spain (ANECA) for researchers and teaching staff.

The journal Medicina Oral, Patología Oral y Cirugía Bucal, which was established in 2000, is the only Spanish journal listed in the JCR and, therefore, it is the journal in which most authors from Spanish universities publish their papers, particularly those from Valencia, Barcelona or Seville. As it is a Spanish journal, the number of citations it receives is limited, which is disadvantageous for the journal itself and for Spanish universities. There is a clear relationship between geographic situation and potential citations. A total of 91 journals were listed in the JCR in 2015, whilst the SJR classification included a total of 170 journals. As in other disciplines, the number of Spanish authors who publish in international journals is increasing (13).

This kind of study has several limitations. The main limitation is related to time, as the results vary depending on which study period is selected and the date on which the search is carried out. Databases also influence the results as they were established in different years (Web of Knowledge emerged in 1950, Scopus in 1996 and Google Scholar in 2004), and have different journals indexed. Web of Knowledge favours Anglo-Saxon journals more than the Scopus database. In the JCR, the only Spanish journal that is indexed is Medicina Oral, Patología Oral y Cirugía Bucal, whilst Scopus lists three together Medicina Oral, Patología Oral y Cirugía Bucal other Spanish journals for the area of dentistry: the Journal of Clinical and Experimental Dentistry (2011), Revista Portuguesa de Estamtología, Medicina Dentaria e Ciurgía Maxilofacial (2008), Revista Española de Cirugía Oral y Maxilofacial (2007), and Avances en Odontoestomatología (2004).

Another limitation is related to the identification of authors (22). It is particularly difficult to analyse Spanish and Asian authors, as stated in other studies. More than one author may have exactly the same name, or one author may have various forms of his/her name or affiliations. This makes it difficult to analyse the database. To eliminate these errors, the search for authors was carried out using the different forms of names included in the database or using the ORCID identifier, when applicable.

## Conclusions

Public universities have the best indexes of output and quality, and the University of Valencia and University Complutense of Madrid obtain the best results. The areas of dentistry that are most developed in the universities are: oral surgery, periodontics, dental materials and oral medicine. The most productive authors are José Vicente Bagán, Cosme Gay Escoda and Manuel Toledano, although those with the highest h-indexes are Manuel Toledano, Mariano Sanz, and Raquel Osorio. The universities’ research models vary. The universities of Barcelona and Valencia, as well as the Complutense University of Madrid, focus their scientific output on very few areas. In contrast, the University of Granada’s production model covers many areas. Universities with more international collaboration had greater visibility and output.
